# ﻿Ultrastructure of three Species of *Entomoneis* (Bacillariophyta) from Lake Qinghai of China, with reference to the external areola occlusions

**DOI:** 10.3897/phytokeys.189.78149

**Published:** 2022-01-25

**Authors:** Ji-Yan Long, David M. Williams, Bing Liu, Wen-Hui Mo, Si-Jin Quan

**Affiliations:** 1 College of Biology and Environmental Sciences, Jishou University, Jishou 416000, China Jishou University Jishou China; 2 Department of Life Sciences, the Natural History Museum, Cromwell Road, London SW7 5BD, UK Department of Life Sciences, The Natural History Museum London United Kingdom

**Keywords:** brackish water, hymen strip, hymen strip region, junction line, valvocopula

## Abstract

Three sympatric *Entomoneis* species, found at the same specific locality in Lake Qinghai, China, are studied by using light and scanning electron microscope. Two species are proposed as new to science and named as *E.sinensis* sp. nov. and *E.qinghainensis* sp. nov. The third species is identified as *E.paludosa* (W. Smith) Reimer. *Entomoneissinensis* has a linear-lanceolate valve outline and Ƨ-shaped keel, bears two distinct 8-shaped loops formed by the valvocopula pars media in each cell and each of its stria is composed of either a long hymen strip or a long hymen strip plus one separated areola close to the raphe. Its hymen strip belongs to Type Two, which is a siliceous membrane strip perforated by two rows of linear pores next to transapical costae and two rows of rounded pores between these two rows of linear pores. *Entomoneisqinghainensis* has large cells, very high keel and evident hymen strip regions like a U-shaped neck pillow at the middle of valve face. Its hymen strip belongs to Type One, which is a siliceous membrane strip perforated by irregularly distributed round pores. *Entomoneispaludosa* also has the hymen strip regions that are worm-like and close to the raphe canal. Its hymen strip is same as that of *E.qinghainensis*. The two kinds of the outside areola occlusions in *Entomoneis* are compared, summarised and discussed.

## ﻿Introduction

Species in the diatom genus *Entomoneis* generally exhibit panduriform frustules in a girdle view and have a sigmoid keel. According to AlgaeBase website (Guiry and Guiry 2021), 30 species names have been accepted taxonomically, based on the listed literature under the species name. Species in this genus occupy a wide range of habitats and exhibit a certain amount of morphological diversity. Before Liu et al. (2018) described the freshwater species *E.triundulata* Bing Liu & D.M. Williams, all species within *Entomoneis* were considered either marine or brackish (Round et al. 1990). Regarding the morphological diversity of *Entomoneis*, some species possess three kinds of fibulae: raphe fibulae, keel fibulae and basal fibulae [e.g. *E.calixasini* Paillès, Blanc-Valleron & Poulin (in Paillès et al. 2014)]. Some possess two kinds of fibulae: raphe fibulae and basal fibulae [e.g. *E.paludosa* (W. Smith) Reimer (in Osada and Kobayasi 1990a; Dalu et al. 2015)] and others have only raphe fibulae [e.g. *E.aequabilis* Osada & Kobayasi (Osada and Kobayasi 1991)]. Some species possess unique characters. For example, *E.centrospinosa* Osada & Kobayasi has 1(0)–3 spines on each side of the central nodule (Osada and Kobayasi 1990b) and *E.annagodheae* Al-Handal & Mucko has an obliquely transapical fascia (Al-Handal et al. 2020a, b). The striae in *Entomoneis* can be uniseriate (e.g. *E.triundulata* and *E.annagodheae*), biseriate [e.g. *E.centrospinosa* and *E.reimeri* Reinke et Wujek (Reinke and Wujek (2013)] or strip-like [e.g. *E.aequabilis*, *E.punctulata* (Grunow) Osada & Kobayasi and *E.pseudoduplex* Osada & Kobayasi (Osada and Kobayasi 1990a)]. Many publications that include scanning electron micrographs often provide only images of low magnification; there are a number of ultrastructural details that require more detailed study (Liu et al. 2018). One example is the outside occlusions of the areolae of *Entomoneis*.

Round et al. (1990) called the very delicate silica membrane which occludes the pores of many raphid diatoms, a hymen, which is perforated by round or elongate pores ca. 5–10 nm in their shortest diameter. Thus, two types of hymenes can be proposed: Type One is perforated by round pores; Type Two is mainly perforated by elongate (linear) pores – both types of hymenes exist in *Entomoneis*. Type One has been noted for *E.paludosa* (Osada and Kobayasi 1990a, p. 170, figs 13–17), Type Two for *E.punctulata* and *E.pseudoduplex* (Osada and Kobayasi 1990a, p. 171, figs 26 and 27; p. 172, figs 38 and 39, respectively) and *E.aequabilis* (Osada and Kobayasi 1991, p. 160, figs. 12 and 13). Round et al. (1990) noted that the whole stria consists of a siliceous membrane in some *Entomoneis* species (e.g. *E.punctulata*, *E.pseudoduplex* and *E.aequabilis*). Osada and Kobayasi (1990a, p. 165) used “hymen-like strips” to describe the whole stria that consisted of a siliceous membrane in *E.pseudoduplex*. However, a part of a whole stria can consist of a hymen-like strip, such as in *E.paludosa* whose valve middle region is composed of these (Osada and Kobayasi 1990a, p. 170, fig. 10). In the current study, we provide high magnification images of the scanning electron microscope for three species of *Entomoneis* found in Lake Qinghai, China with the aim of illustrating both types of hymen occlusions.

Lake Qinghai is the largest inland brackish-water lake in China. Its diatom flora has been investigated since 1979 (e.g. Lanzhou Institute of Geology and Chinese Academy of Sciences 1979; Yao et al. 2011; Peng et al. 2013). These studies have provided a list of taxa, but lack useful illustrations (drawings or micrographs) for the taxa observed. However, some interesting new species recently published have been well documented (Peng et al. 2014; 2016; Liu et al. 2020; Deng et al. 2021), confirming that endemic diatom species inhabit this ancient lake. This paper provides further evidence of endemic taxa with the description of two new species of *Entomoneis*.

## ﻿Materials and methods

### ﻿Site description

Three sampling sites were chosen from the lakeshore waters of Lake Qinghai (see Liu et al. 2020, p. 116, fig. 1). Geographically, Lake Qinghai is located between longitudes 99°36' and 100°47', latitudes 36°32' and 37°15' in Qinghai Province, China. It is the largest inland brackish-water lake in China. The Lake has ca. 4294 km^2^ surface water area and is ca. 3200 m a.s.l. Its climate belongs to the plateau-continental climate. The average annual temperature is ca. -0.7 °C, the ranges of the average annual precipitation and the average annual evaporation in the Lake region are 319–395 mm and 800–1000 mm, respectively for many years (Luo et al. 2017). More than 50 rivers/streams run into Lake Qinghai and there is no outlet to discharge the Lake water as Lake Qinghai is hydrologically closed. The surface water evaporating is nearly the sole path for loss of lake water. The lake has an 18.3 m average water depth and maximum of 26.6 m; the average values for alkalinity and pH are 25.6 mmol l^·1^ and 9.2 respectively (Peng et al. 2014). There is a three-month ice-covered period (middle November to middle February) in Lake Qinghai so that the growth period for diatoms is mainly from May to October.

### ﻿Sampling

At the three sampling sites in Lake Qinghai (see Liu et al. 2020, p. 116, fig. 1), there are numerous submerged stones with yellow-brown surfaces which indicate many diatoms growing on them. Each stone sampled was placed on a plastic plate, then its surfaces brushed using a toothbrush, with the brushed-off diatom samples being washed on to the plate. The samples were transferred to a 100 ml sampling bottle and fixed with 70% ethanol. Two bottle diatom samples were collected for each sampling site. Together with the sample collection, temperature, pH and conductivity were measured in situ with a portable multimeter (HQ40D, HACH Company).

### ﻿Methods

The samples were processed (cleaned of organic material) for microscope examination using 10% hydrochloric acid (HCl) and 30% hydrogen peroxide (H_2_O_2_). Permanent slides were prepared using the Mounting Medium Naphrax. These slides were examined and the specimens photographed, using a Leica DM3000 light microscope (LM) and a Leica MC190 HD digital camera. The holotype slides are deposited in the Natural History Museum, London, United Kingdom (BM) and isotype slides are kept in the Herbarium of Jishou University, Hunan, People’s Republic of China (JIU).

Samples were further examined using a scanning electron microscope (SEM). Several drops of the selected cleaned diatom material were air-dried on to glass coverslips. Coverslips were attached to aluminium stubs using double-sided conductive carbon strip and sputter-coated with platinum (Cressington Sputter Coater 108auto, Ted Pella, Inc.). Samples were examined and imaged using a field emission scanning electron microscope (FE-SEM) Sigma HD (Carl Zeiss Microscopy) available at Huaihua University, China.

Diatom terminology largely follows Ross et al. (1979), Paddock and Sims (1981) and Round et al. (1990), specifically for species in *Entomoneis*, Osada and Kobayasi (1985, 1990a) were followed. We have proposed two types of hymenes, hymen strip and hymen strip region for *Entomoneis* (see below section Discussion, Fig. 14).

## ﻿Results

### Class Bacillariophyceae Haeckel

**Order Surirellales D.G. Mann**

**Family Entomoneidaceae Reimer**

**Genus *Entomoneis* Ehrenberg**

#### ﻿ Entomoneis sinensis

Taxon classificationPlantaeSurirellalesEntomoneidaceae

Bing Liu & D.M. Williams sp. nov.

26D05101-F45C-55CD-AFDE-C8AA9CBD0D44

Figs 1 , 2 , 3 , 4 , 5 , 6 , 7 , 14

##### Holotype.

**Figure 1.**
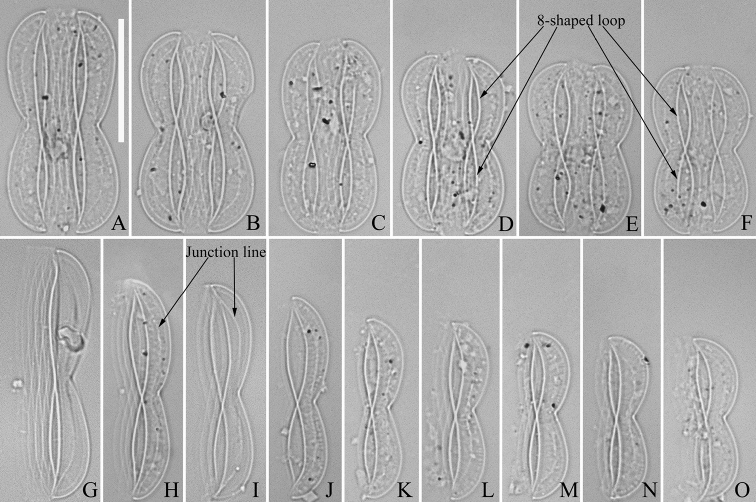
**A–O***Entomoneissinensis* sp. nov., girdle view, LM**A–F** six frustules showing a size diminution series, note the distinctive 8-shaped loops (labelled in Figs **D–F**) **G–O** nine valves showing a size diminution series, note arcuate junction line (labelled in Figs **H** and **I**) **A** micrograph of holotype specimen **B** micrograph of isotype specimen. Scale bar: 20 μm.

Slide BM 81941, the holotype specimen circled on the slide, illustrated here as Fig. 1A; isotype, slide JIU202101, illustrated here as Fig. 1B.

##### Type locality.

China. Qinghai Province, Lake Qinghai, a sampling point near the lakeshore, 36°50'34" N, 99°42'39" E, 3210 m a.s.l., collected by Bing Liu, 19 July 2019.

**Figure 2.**
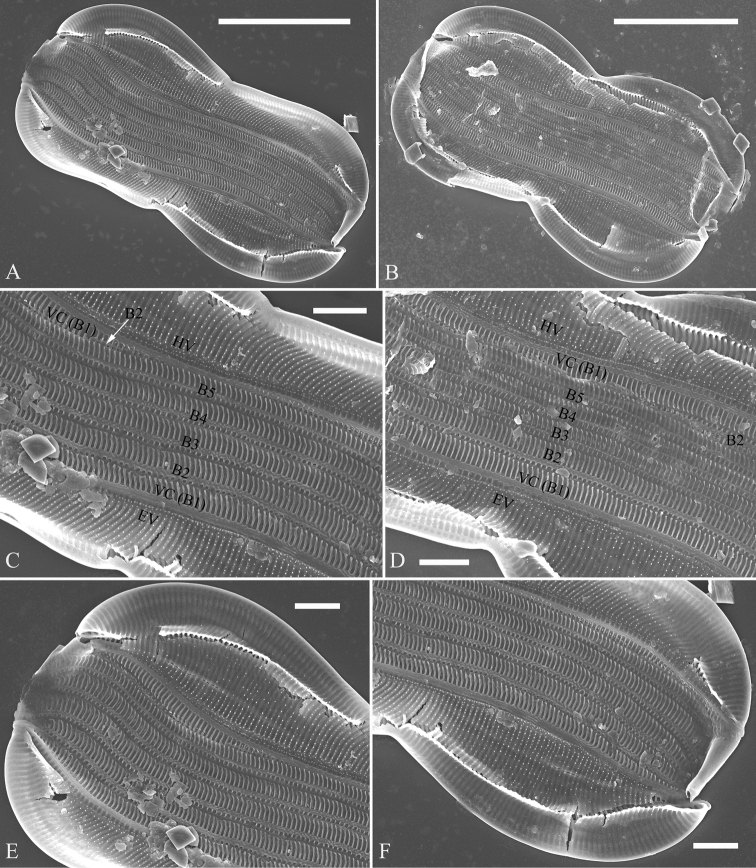
**A–F***Entomoneissinensis* sp. nov., girdle view, SEM**A, B** two frustules, note sigmoid girdle bands **C, D** central parts from Fig. **A** and **B** respectively, note the 5:2 configuration of girdle bands, i.e. five girdle bands, B1(Valvocopula, VC) to B5, associated with epivalve (EV); two girdle bands, B1(Valvocopula, VC) and B2, associated with hypovalve (HV) **E, F** two apices from Fig. **A**. Scale bars: 10 μm (**A, B**); 2 μm (**C–F**).

##### Description.

***LM*** (Fig. 1). Frustule panduriform in girdle view (Fig. 1A–F). Frustule dimensions (n = 41): length 22.6–42.6 μm, width 8.9–14.1 μm at its centre, 14.6–19.8 μm at its widest region. Two distinct 8-shaped loops are present in each frustule (indicated in Fig. 1D–F), one 8-shaped loop evident in each valve (Fig. 1G–O). Simple, arcuate junction line discernible in some specimens (indicated in Fig. 1H and I). Costae and striae invisible under LM. Girdle bands numerous.

**Figure 3.**
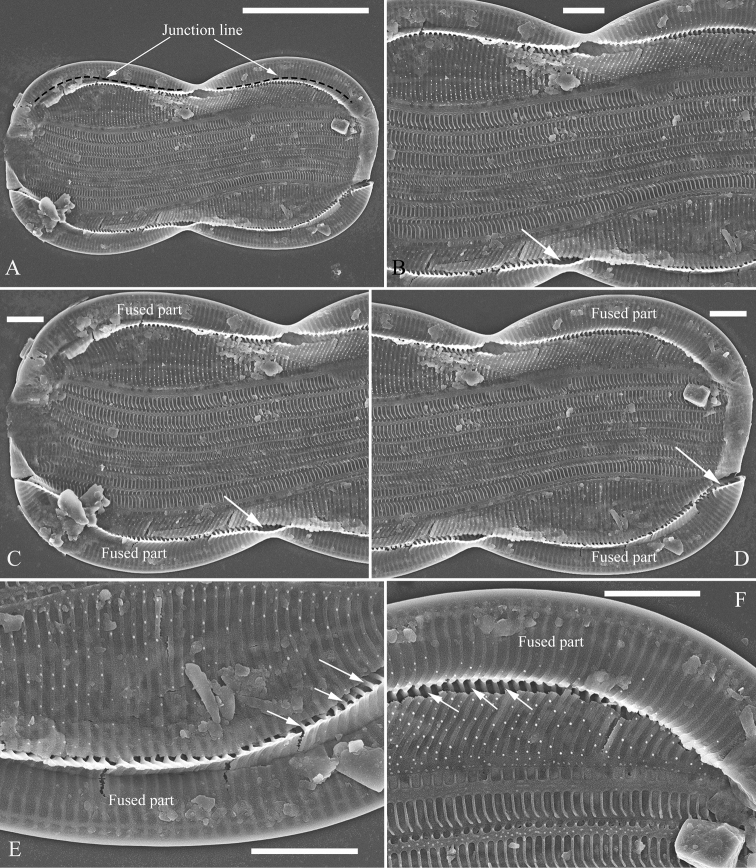
**A–F***Entomoneissinensis* sp. nov., girdle view, SEM**A** one broken frustule, note the simple arcuate junction lines **B–D** details from Fig. **A** note the fused parts of two sides of the keel and the subraphe canal connecting the cell lumen only near the central (Figs **B** and **C**, arrow, respectively) and the distal raphe ending (Fig. **D**, arrow) **E, F** details from Fig. **A** note the short, bar-like basal fibulae (three arrows, respectively). Scale bars: 10 μm (**A**); 2 μm (**B–F**).

**Figure 4.**
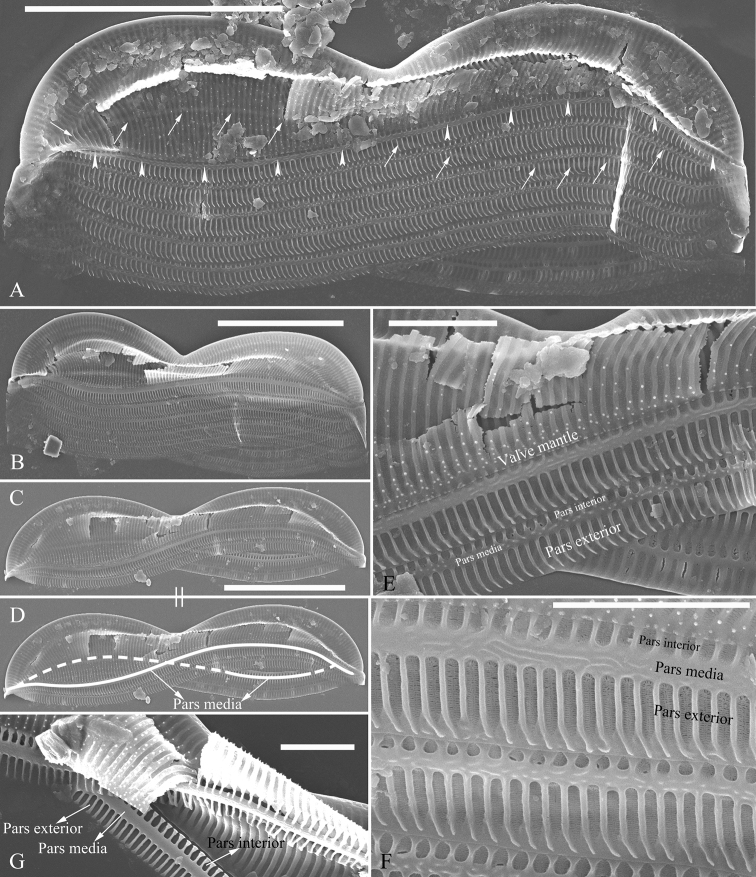
**A–G***Entomoneissinensis* sp. nov., SEM**A** one valve with associated girdle bands, note the 8-shaped loop formed by the pars media of valvocopula (the pars media indicated by arrowheads and arrows indicating the pars media on the unseen side) **B–D** two valves with girdle bands further showing the 8-shaped loop (labelled in Fig. **D**) **E** middle part of a valve, note the mantle and the three parts of each girdle bands: parts exterior, pars media and pars interior **F** detail from Fig. **E** note the hymen strip occluding the poroid **G** valvocopula in internal view, note the broader pars exterior than pars interior and sternum-like pars media. Scale bars: 10 μm (**A–D**); 2 μm (**E–G**).

***SEM*, *girdle view*** (Figs 2–4). Frustule panduriform with low keel (Fig. 2A and B; Fig. 3A–F, indicated by fused part). Cells having a 5:2 configuration of girdle bands, i.e. five girdle bands associated with the epivalve and two associated with the hypovalve (Fig. 2C–F, labelled in Fig. 2C and D). Junction line simple, arcuate (Fig. 3A). Two sides of the keel fused so that subraphe canal connects the cell lumen only near the central ending (Fig. 3B and C, arrow, respectively; see also Fig. 7B, arrow) and distal raphe ending (Fig. 3D, arrow). Short bar-like basal fibulae forming junction line (Fig. 3E and F, three arrows, respectively). Each pars media of valvocopula forming 8-shaped loop that is very distinct under LM (Fig. 4A–D). Each costa extending from raphe canal to inconspicuous mantle, warts bearing on each costa (Fig. 4E). Structure of each girdle bands similar, composed of pars exterior, pars media and pars interior (Fig. 4E). Pars media like a sternum, not located at the mid-line but slightly displaced towards pars interior (Fig. 4G). Both pars exterior and interior composed of one row of elongate poroids and each elongate poroid consisting of two costae and a hymen strip between them (thus the elongate poroid is n-shaped), with the n-shaped poroid of pars exterior longer than that of pars interior (Fig. 4F and G).

**Figure 5.**
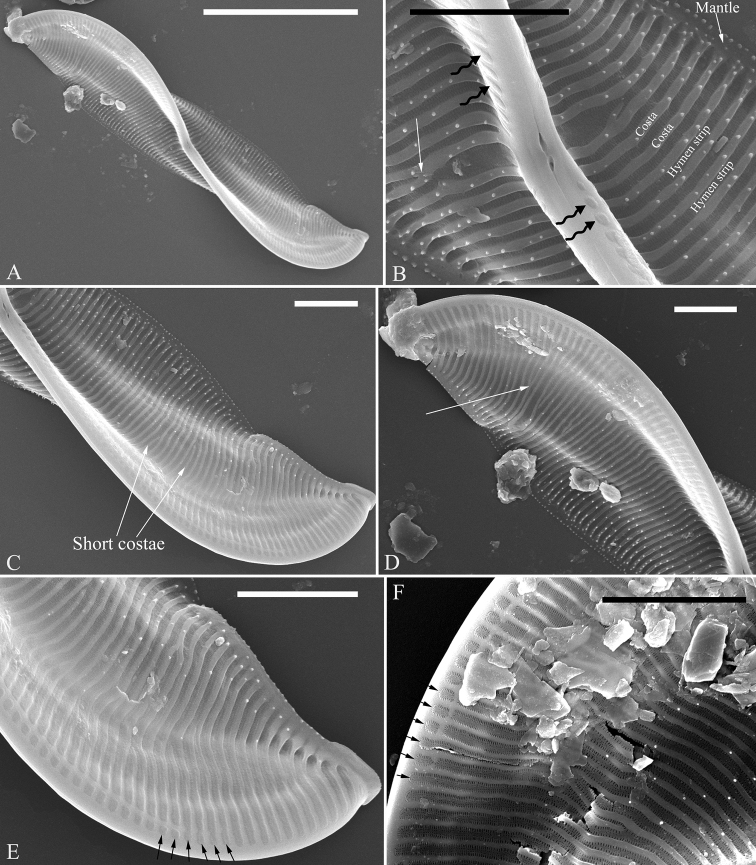
**A–F***Entomoneissinensis* sp. nov., valve external view, SEM**A** one whole valve showing the Ƨ-shaped keel outline **B** central part from Fig. **A** note hymen strips, costae, mantle, warts, forked costa (arrow) and one separated row of rounded areolae at each side of the raphe (wavy arrows) **C, D** two apices from Fig. **A** note the short costae (two arrows in Fig. **C**) and two costae merging into one (arrow in Fig. **D**) **E, F** details showing one separated row of rounded areolae terminating before the apex (six arrows, respectively). Scale bars: 10 μm (**A**); 2 μm (**B–F**).

**Figure 6.**
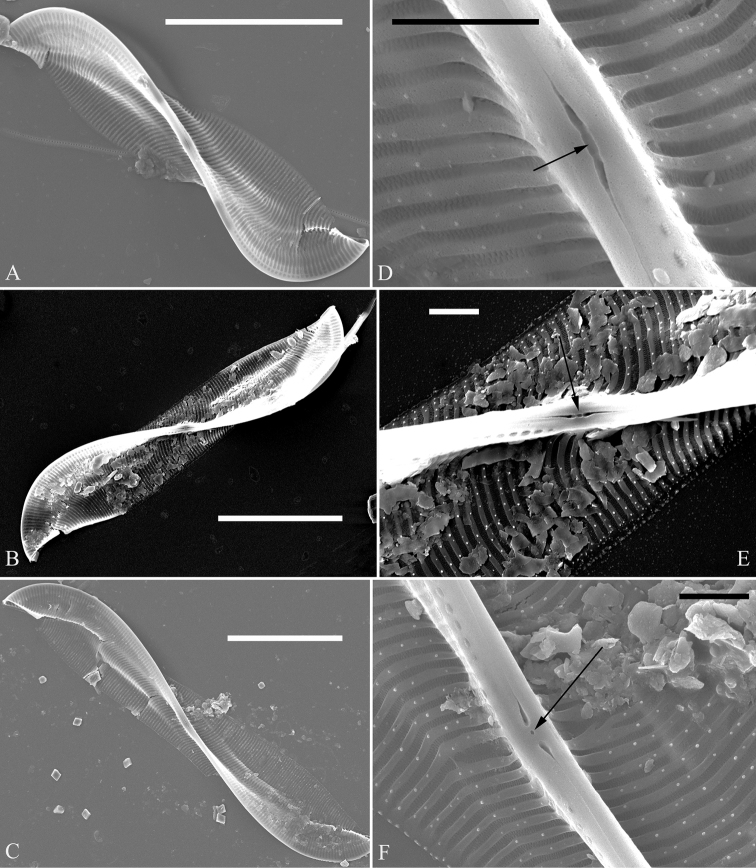
**A–F***Entomoneissinensis* sp. nov., external view, SEM**A–C** three whole valves, note Ƨ-shaped keel outline **D–F** three middle parts from Figs **A, B** and **C** respectively, note two proximal raphe endings slightly dilated and a pore-like structure at the centre of central nodule (arrows). Scale bars: 10 μm (**A–C**); 1 μm (**D–F**).

**Figure 7.**
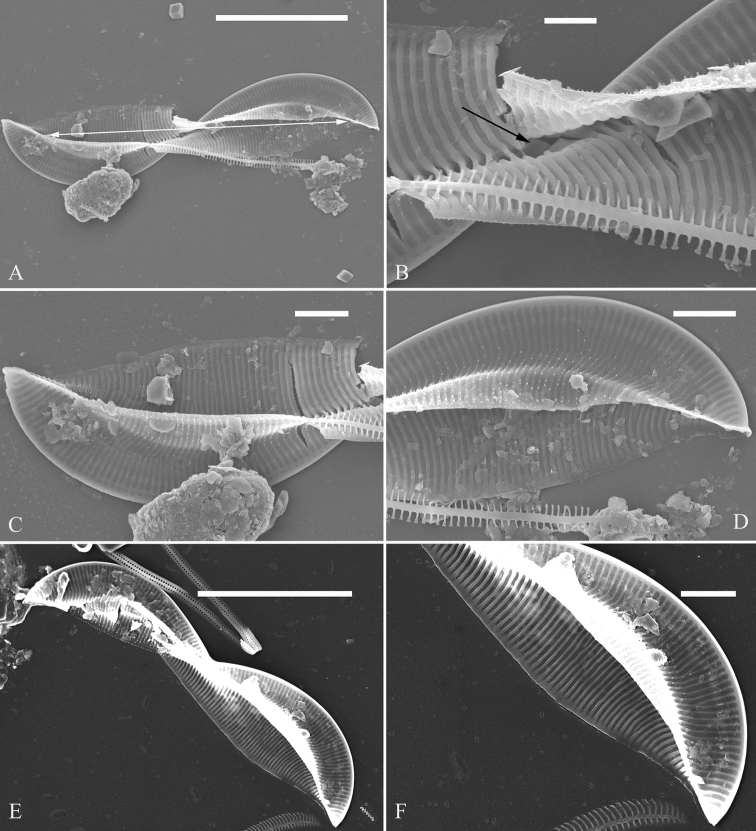
**A–F***Entomoneissinensis* sp. nov., valve internal and side views, SEM**A** one valve showing only one lumen (no sub-compartments present) in a cell (double-headed arrow) **B** middle part from Fig. **A** note the canal raphe communicates with the cell interior at the valve centre **C, D** two apices from Fig. **A E, F** another valve in side view, note the cell lumen. Scale bars: 10 μm (**A, E**); 2 μm (**B–D, F**).

***SEM*, *valve view*** (Figs 5–7). Valve linear-lanceolate, keel Ƨ-shaped (Fig. 5A, Fig. 6A–C). Costae mostly running from raphe canal to mantle (Fig. 5B), but some bifurcate (Fig. 5B, arrow), some terminating halfway to mantle (Fig. 5C, short costae). Each stria included between two adjacent costae, composed of one hymen strip (Fig. 5A–F). This type of hymen strip belonging to Type Two hymen strip, which is a siliceous membrane strip perforated by two rows of elongate (linear) pores next to the transapical costae and two rows of rounded pores between these two rows of linear pores (Fig. 14A and B). Mantle inconspicuous (Fig. 5B). Two total rows of separated areolae present along the raphe on both sides of the keel (Fig. 5B, four wavy arrows), but do not extend to the apex (Fig. 5E and F, six arrows, respectively). The hymenes occluding these separated areolae have the same structure as the hymen strip on striae (Fig. 5F, arrows). Stria density 36–43 in 10 μm (n = 7). Two proximal raphe endings slightly dilated and a pore-like structure located at the centre of central nodule (Fig. 6D–F, arrow, respectively). Internally, one cell bearing only one lumen, no sub-compartment present (Fig. 7).

##### Etymology.

Named after China where the species was found (the specific locality is Lake Qinghai).

##### Ecology and distribution.

*Entomoneissinensis* was found on the stone surfaces in Lake Qinghai. The following environmental parameters were measured in the field. Conductivity was 16296.7 ± 86.2 μS/cm, pH was 9.14 ± 0.01 and water temperature was 15.5 ± 0.3°C. According to above data and because Lake Qinghai is a brackish water lake, *Entomoneissinensis* should be a brackish water diatom species. So far, *E.sinensis* is only found in the type locality and is a dominant species.

#### ﻿ Entomoneis qinghainensis

Taxon classificationPlantaeSurirellalesEntomoneidaceae

Bing Liu & D.M. Williams sp. nov.

F21AE59D-5058-505E-8C65-EB5037E41035

**Figure 8.**
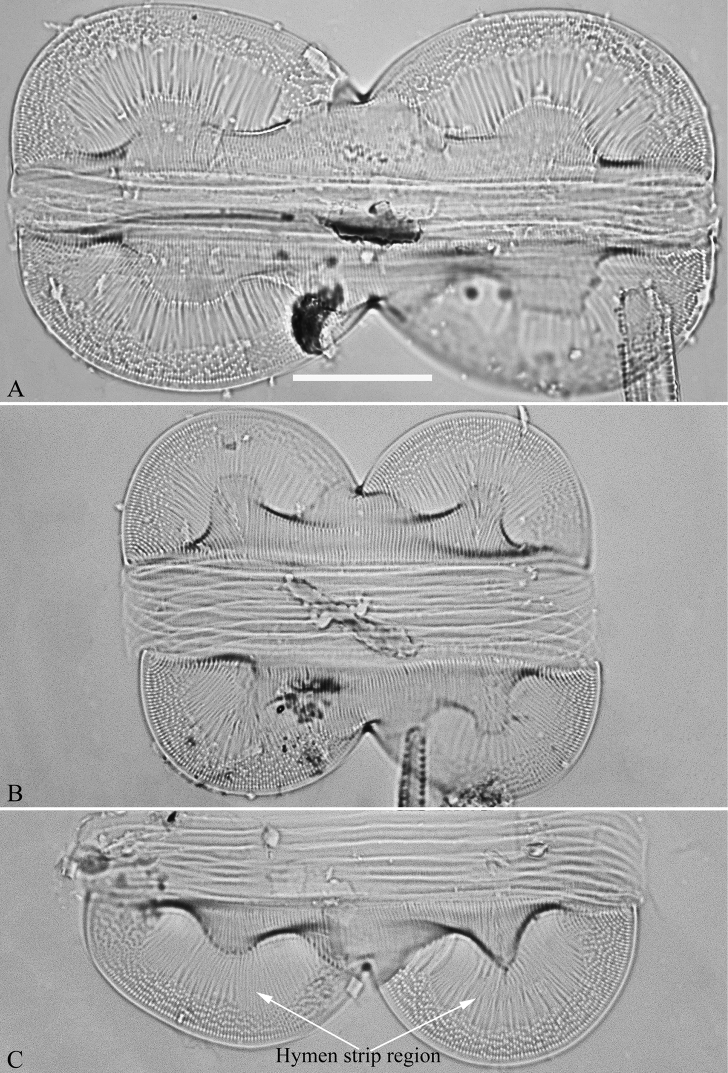
**A–C***Entomoneisqinghainensis* sp. nov., girdle view, LM**A, B** two whole frustules, note the distinctive hymen strip region (labelled in Fig. **C**) **C** epivalve with associated girdle bands, note the hymen strip region and junction line **A** micrograph of holotype specimen **B** micrograph of isotype specimen. Scale bar: 20 μm.

**Figure 9.**
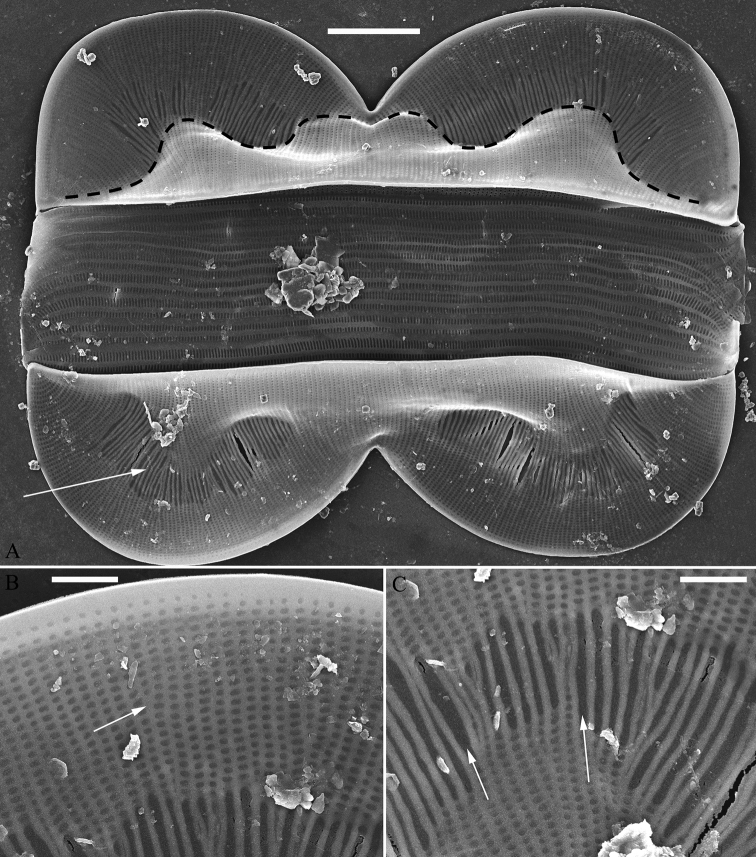
**A–C***Entomoneisqinghainensis* sp. nov., frustule view, SEM**A** one complete frustule, note the undulate junction line (indicated by dotted line) and the hymen strip region **B** detail from Fig. **A** note the striae composed of many single areolae near the sternum and two costae merging into one (arrow) **C** detail from Fig. **A** note hymen strips (two arrows). Scale bars: 10 μm (**A**); 2 μm (**B, C**).

**Figure 10.**
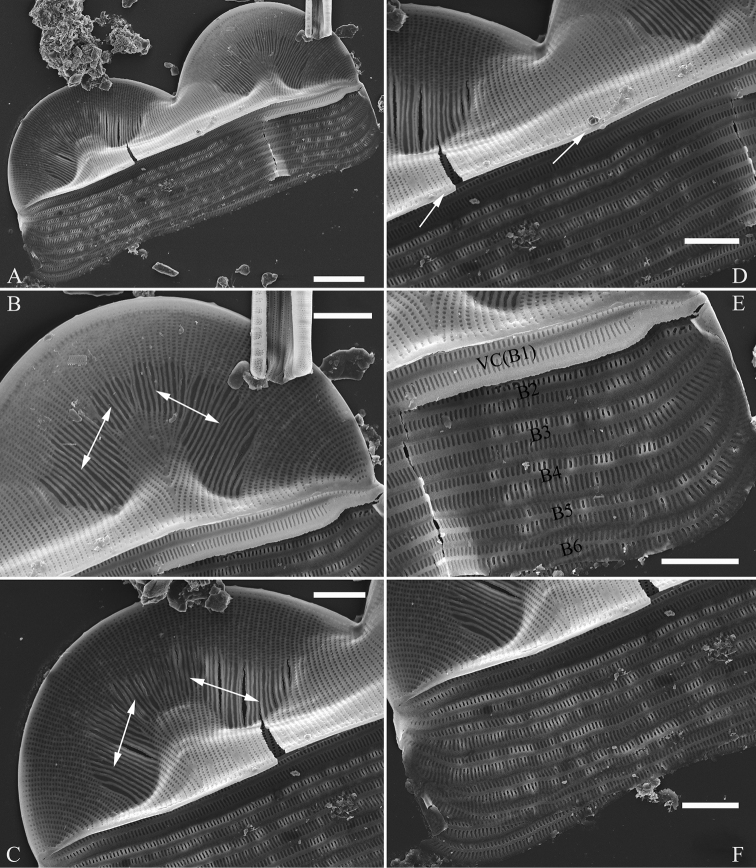
**A–F***Entomoneisqinghainensis* sp. nov., girdle view, SEM**A** оne valve with numerous girdle bands **B, C** two apices from Fig. **A** note the hymen strip regions (two double-headed arrows, respectively) **D** central part from Fig. **A** note thickened mantle **E, F** two girdle details from Fig. **A** note at least six girdle bands (labelled B1 to B6) associated with the epivalve. Scale bars: 10 μm (**A**); 5 μm (**B–F**).

Figs 8 , 9 , 10 , 14

##### Holotype.

Slide BM 81942, the holotype specimen circled on the slide, illustrated here as Fig. 8A; isotype, slide JIU202102, illustrated here as Fig. 8B.

##### Type locality.

China. Qinghai Province, Lake Qinghai, a sampling point near the lakeshore, 36°50'34" N, 99°42'39" E, 3210 m a.s.l., collected by Bing Liu, 19 July 2019.

##### Description.

***LM*** (Fig. 8). Frustules panduriform in girdle view (Fig. 8A and B). Frustule dimensions (n = 19): length 67.1–116.5 μm, width 21.6–37.5 μm at middle constricted part, 46.6–62.5 μm at widest part. Keel very high. Hymen strip region distinct, like a U-shaped neck pillow, located at the middle of each lobe of valve (indicated in Fig. 8C). Junction line sinuous with a distinct bulge into the hymen strip region (Fig. 8, see also Fig. 9A). Striae visible under LM, 18–23 in 10 μm. Girdle bands numerous.

***SEM*, *girdle view*** (Figs 9 and 10). Frustule panduriform, composed of epivalve, girdle bands and hypovalve (Fig. 9A). Junction line confirmed same as LM observation (Fig. 9A, dotted line). Costae mostly running from raphe canal to mantle, but sometimes two costae merging into one (Fig. 9B, arrow). Hymen strip region composed of costae and hymen strips (Fig. 9A and C, two arrows, respectively; Fig. 10B and C, two double-headed arrows, respectively). This type of hymen strip belongs to Type One hymen strip, which are a siliceous membrane strip perforated by irregularly distributed round pores (Fig. 14C and D). Mantle thickened (Fig. 10B, two arrows). Six girdle bands associated with epivalve (Fig. 10E, labeled B1 to B6). Structure of each girdle bands similar, composed of pars exterior, pars media, and pars interior. The poroids of each girdle band elongate (Fig. 10F).

##### Etymology.

Named after Lake Qinghai, where the species was found.

##### Ecology and distribution.

*Entomoneisqinghainensis* was commonly found on the stone surfaces in Lake Qinghai with *E.sinensis*. According to above data and because Lake Qinghai is a brackish water lake, *E.qinghainensis* should be a brackish water diatom species. So far, *E.qinghainensis* is only found in the type locality and is not a dominant species.

#### ﻿ Entomoneis paludosa

Taxon classificationPlantaeSurirellalesEntomoneidaceae

(W. Smith) Reimer

71E15944-5BCB-503B-AB3B-6DFA6CB44FCA

**Figure 11.**
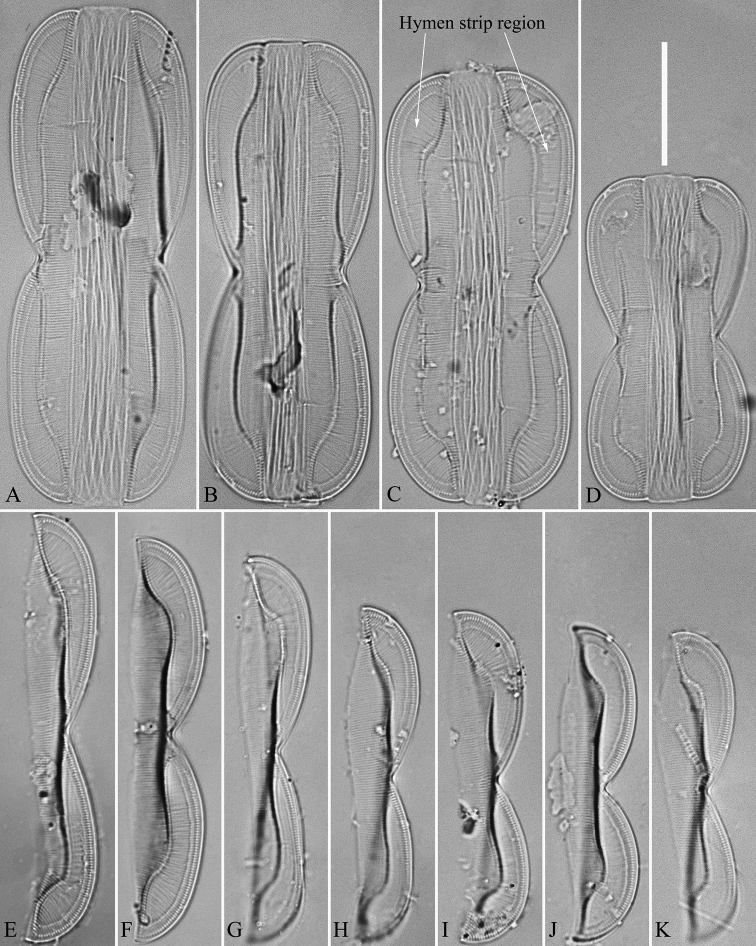
**A–K***Entomoneispaludosa*, girdle view, LM**A–D** four complete frustules, note the hymen strip region (indicated in Fig. **C**) **E–K** seven valves in side view showing size reduced series. Scale bar: 20 μm.

**Figure 12.**
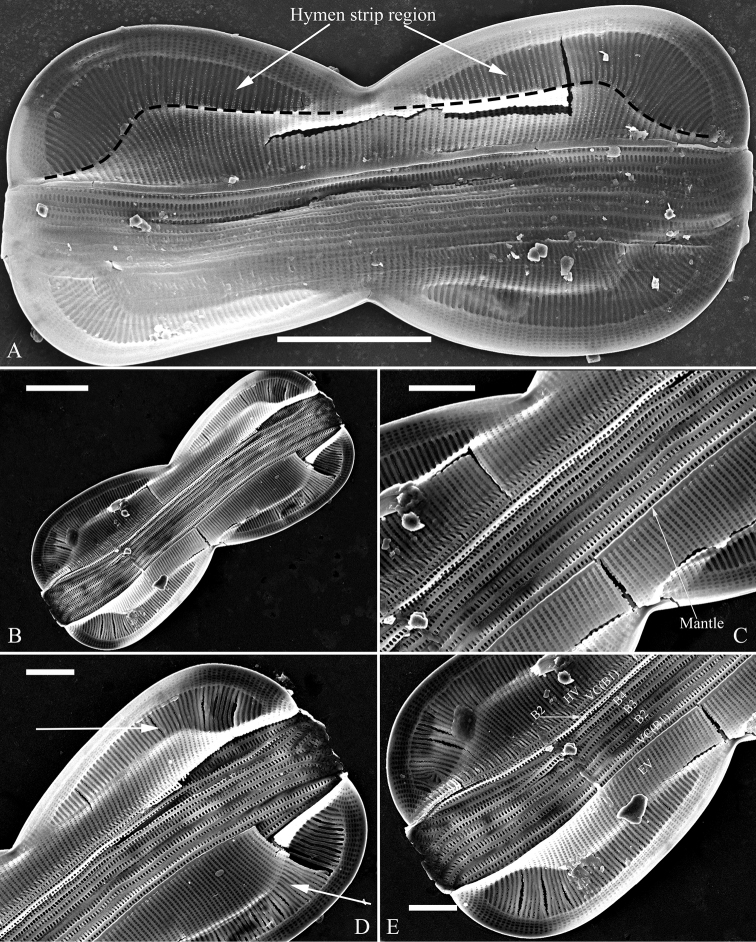
**A–E***Entomoneispaludosa*, girdle view, SEM**A** one whole frustule, note the worm-like hymen strip region and the junction line (indicated by the dotted line) **B** another frustule **C–E** details from Fig. **B** note the thickened mantle (Fig. **C**, arrow), worm-like hymen strip region (Fig. **D**, two arrows) and 4:2 configuration of the girdle bands (i.e. four girdle bands associated with epivalve (EV) and two associated with hypovalve (HV) (Fig. **E**). Scale bars: 10 μm (**A, B**); 2 μm (**C–E**).

**Figure 13.**
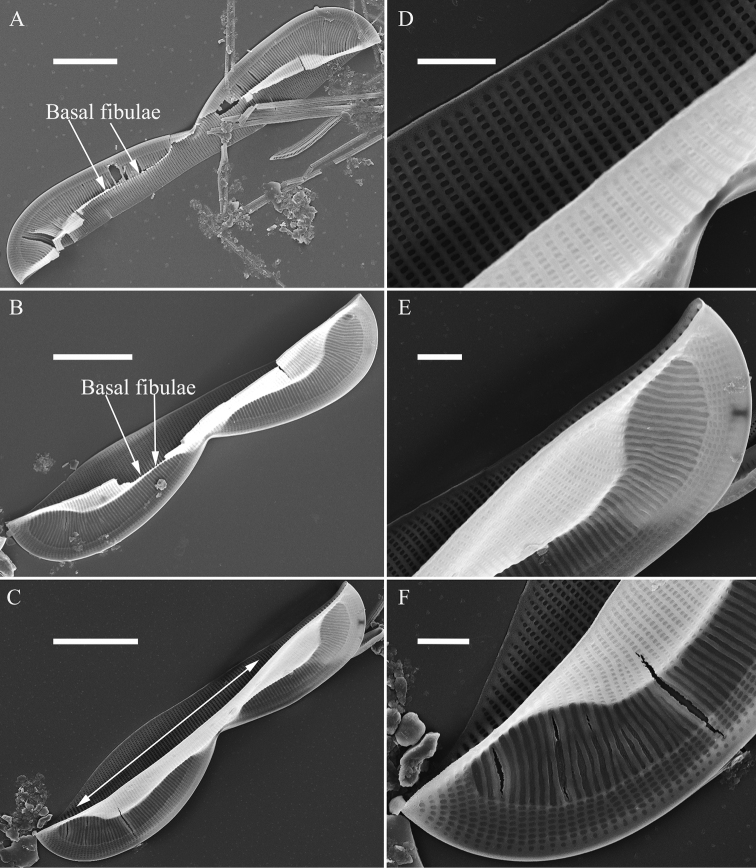
**A–F***Entomoneispaludosa*, valve side view, SEM**A–C** three valves in side view, note the basal fibulae and the frustule cavity without sub-compartments (indicated by double-headed arrow) **D–F** details from Fig. **A–C**. Scale bars: 10 μm (**A–C**); 2 μm (**D–F**).

**Figure 14.**
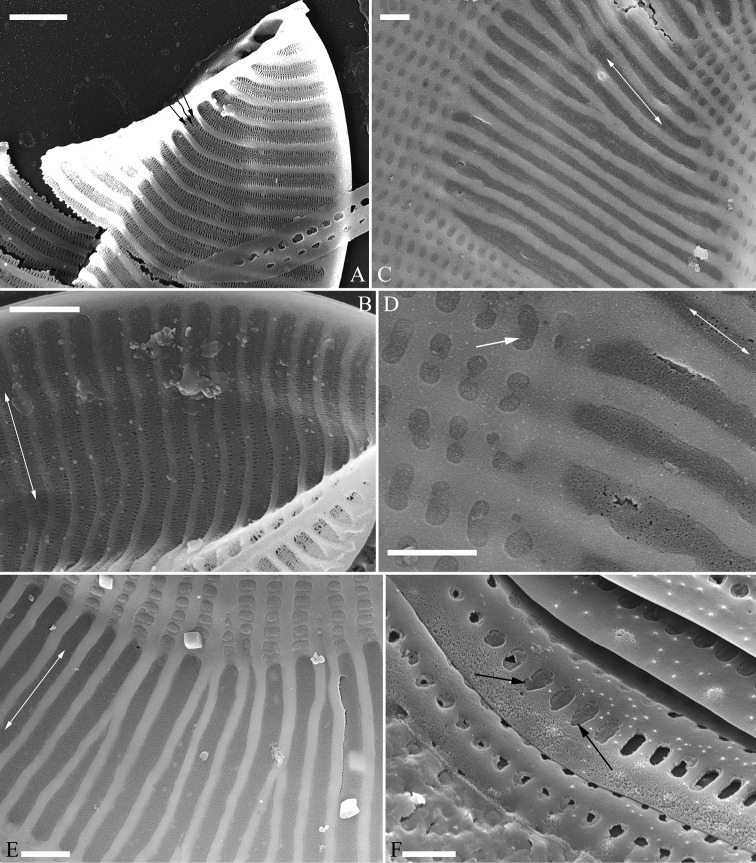
**A–F** Details of hymen and hymen strip in *Entomoneis*, SEM**A, B** details of hymen strip of Type Two from *E.sinensis* sp. nov., note the two rows of linear pores (Fig. **A**, two arrows) on either side of the two intermittent rows of rounded pores (Fig. **A**, wavy arrow) **C, D** details of hymen strip of Type One from *E.qinghainensis* sp. nov., note the hymen is finely perforated over both the areolae (Fig. **D** arrow) and the hymen strips (Fig. **D**, double-headed arrow) **E** detail of hymen strip of Type One from *E.paludosa*, note the hymen is finely perforated over both the areolae and the hymen strips (double-headed arrow) **F** detail of hymenes from *E.triundulata*, note the hymenes of the areolae in the girdle bands (two arrows). Scale bars: 600 nm (**A–F**).

Figs 11 , 12 , 13 , 14

##### Observation.

***LM*** (Fig. 11). Frustules panduriform in girdle view (Figs 11A–D). Frustule dimensions (n = 19): length 34.2–80.7 μm, width 12.8–21.8 μm at constricted part, 22.4–30.3 μm at widest part. Keel high. Hymen strip region distinct, worm-like (i.e. curved from apex to valve centre, widest near distal end), located close to the raphe canal (Fig. 11A–D, indicated in Fig. 11C, see also Fig. 12A, B and D). Junction line slightly sinuous (Fig. 11, see also Fig. 12A). Striae visible under LM, 22–25 in 10 μm. Girdle bands numerous.

***SEM*** (Figs 12 and 13). Frustule panduriform, composed of epivalve, girdle bands and hypovalve (Fig. 9A and B). Junction line confirmed same as LM observation (Fig. 12A, dotted line). Costae mostly running from raphe canal to mantle. Hymen strip region composed of costae and hymen strips (Fig. 12A and D, two arrows, respectively). This type of hymen strip belongs to Type One hymen strip, which are a siliceous membrane strip perforated by irregularly distributed round pores (Fig. 14E). Mantle thickened (Fig. 12C, arrow). Cells having a 4:2 configuration of girdle bands, i.e. four girdle bands associated with epivalve and two associated with hypovalve (Fig. 12C–E, labelled in Fig. 12E). Fibulae having only two levels: raphe fibulae and basal fibulae (Fig. 13A and B, two arrows, respectively). Internally, one cell bearing only one lumen, no sub-compartment present (Fig. 13C–F).

##### Ecology and distribution.

*Entomoneispaludosa* was commonly found on the stone surfaces in Lake Qinghai with *E.sinensis* and *E.qinghainensis* and it has a global distribution. *Entomoneispaludosa* is a dominant species in the sampling sites.

## ﻿Discussion

Liu et al. (2018) summarised the kinds of keel found in species of the genus *Entomoneis*. They suggested that *Entomoneis* exhibited two kinds of keel: S-shaped and Ƨ (reverse)-shaped. Like most species of *Entomoneis*, *E.sinensis* has Ƨ-shaped keel (Fig. 6A–C). *Entomoneissinensis* has strongly sigmoid girdle bands so that in girdle view, each girdle band crossing appearing decussate (Fig. 2A and B, Fig. 4A and B). Using LM, each valve bears a distinct 8-shaped loop which is formed by the two halves of valvocopula pars media (Fig. 4A–C). No other species in *Entomoneis* bear the 8-shaped loop, so it is considered a unique character. *Entomoneispunctulata*, *E.pseudoduplex* and *E.aequabilis* all have whole striae (sometimes with a separated single areola near the raphe) occluded by a siliceous strip membrane (Osada and Kobayasi 1990a, 1991). This strip membrane is one type of hymen (Type Two, according to Round et al. 1990). *Entomoneissinensis* also bear the Type Two hymen strip and here we provide a detailed description: hymen strip of Type Two is a siliceous strip membrane perforated by two rows of linear pores next to transapical costae and two rows of rounded pores between these two rows of linear pores (Fig. 14A and B). The separated areolae close to the raphe (Fig. 5B, wavy arrows) has also this Type Two hymen (see Fig. 5F).

*Entomoneisqinghainensis* and *E.paludosa* are similar in many respects. Most important is that they both possess two hymen strip regions in a valve (see Fig. 9A and Fig. 12A). This hymen strip belongs to Type One, which is a siliceous strip membrane perforated by irregularly distributed round pores (Figs 14C, D and E). These strip hymen regions are positioned between two valve regions consisting of rows of single areolae. So far, there are no other species of *Entomoneis* that possess this kind of strip hymen region. *Entomoneisqinghainensis* can be easily distinguished from *E.paludosa* by the shape of the hymen strip region: the former’s hymen strip region is like a U-shaped neck pillow and located nearly at the middle of the valve surface (further away from the raphe canal, see Fig. 9A), whereas the latter’s is worm-like (i.e. curved from the apex to the valve centre, widest near the distal end, see Fig. 12A, see also Osada and Kobayasi 1990a, fig. 10; Dalu et al. 2015, figs. 2–8) and located close to the raphe canal (not at the middle of the valve surface). Furthermore, the keel of *E.qinghainensis* is higher than that of *E.paludosa* and the stria density of *E.qinghainensis* is lower than that of *E.paludosa* (18–23 vs. 22–25 in 10 μm).

The girdle bands are numerous in the three species described in this paper. *Entomoneissinensis* has a 5:2 configuration of girdle bands, i.e. five girdle bands associated with the epivalve, two associated with the hypovalve (see Figs 2C–F). *Entomoneisqinghainensis* has six girdle bands associated with the epivalve (see Fig. 10). *Entomoneispaludosa* has a 4:2 configuration of girdle bands, i.e. four girdle bands associated with the epivalve and two associated with the hypovalve (see Fig. 12). The girdle band poroids have the same hymen structure as those in the valve.

## Supplementary Material

XML Treatment for Entomoneis sinensis

XML Treatment for Entomoneis qinghainensis

XML Treatment for Entomoneis paludosa
